# Communicating in Verse: How Reading Poetry Can Expand How We Care for Patients

**DOI:** 10.1007/s10912-024-09898-2

**Published:** 2024-10-12

**Authors:** Tulsi R. Patel, Joshua Hauser

**Affiliations:** 1 University of California San Diego, San Diego, CA USA; 2 Jesse Brown VA Medical Center and Northwestern University Feinberg School of Medicine, Chicago, IL USA

**Keywords:** Poetry, Medical humanities, Medical education, Medical students, Poetry curricula

## Abstract

It is a foundational principle of medical humanities that the appreciation of literature and other humanities enrich and expand medical training. But what are the mechanisms by which this happens? As a faculty member and former medical student, who met during an interdisciplinary and multi-institutional 5-session poetry seminar, we reflect on how poetry enriches our own experiences working with patients, as well as how we care for patients. Reading poetry in medicine has the potential to enhance observational skills, model an appreciation of uncertainties, and generate joy. Similar to our seminar, other institutions may also consider incorporating poetry into curricula.

Through a 5-session medical humanities seminar, we (JH) teach poetry and medicine to medical students (TP) at Northwestern University Feinberg School of Medicine. The course is interdisciplinary in both its faculty (including physicians like JH, poets, and a librarian) and audience: in addition to the medical students like TP, we have opened the course virtually to nursing and Physician Assistant (PA) students at two other universities, Harvard and Yale.

All graduate health professional students have a seminar of their choosing as a mandatory part of the university curricula. For those who choose this particular seminar (titled “Poetry & Medicine”), the first class creates some “ground rules” established by participants to create a supportive atmosphere conducive to meaningful dialogue. Our seminar operates on the principle of “brave space,” where students are encouraged to engage deeply and authentically and respect each others’ perspectives. Confidentiality is also vital—stories shared in the seminar are not discussed elsewhere. The final ground rule is to assume the validity of all responses to reading the poems, thereby understanding that there are no “correct” interpretations or right answers. This is inspired by one of the first pieces read in the seminar, where poet Mark Doty remarked about the “pleasure when people talk about poems together as long as nobody is saying, ‘I know what this means and you don’t,’ [but rather] ‘The meaning is what we make right now out of what we read’” (Campo and Doty 2018).

The design of the course is straightforward: one session introducing the overall topic of poetry and medicine and discussing examples of “medical” and “non-medical” poems; two sessions led by local poets reading their work and the works of others; a session describing our experiences using poetry with patients (Segar et al. 2021); and finally a session with staff from the Poetry Foundation about the foundation’s activities, a conversation about the role of poetry in diverse settings such as medicine, and a brief student exercise in writing and sharing it amongst ourselves. With 15 poems as part of the curriculum, some examples of the medically themed ones are “The Chart” by Rafael Campo and “Cancer” by C. Dale Young. Our silent individual reflections followed by collective analyses of “The Chart,” for example, prompted a discussion on how poetry lends correcting voices to false narratives, adds dimensions to our understanding of patients’ inner lives, and illuminates health disparities. The non-medical poems include “Fall” by Mary Oliver, “Keeping Quiet” by Pablo Neruda, and “Facing It” by Yusef Komunyakaa, sparking exercises in close reading, discussions about the many possible interpretations of metaphorical language, and poetry as a form of coping mechanism and reflection.

The unique strengths in the interprofessional nature of the seminar’s students are particularly rewarding. It allows students further practice in collaborating with new individuals, actively listening, and contributing to an intellectual environment, all of which are applicable skills in clinical settings. Students have also found it meaningful to learn alongside future team members in an environment of collective growth. One student had previously remarked, “I really enjoyed this aspect of the class, albeit I was one of the students from the other disciplines. From my perspective I feel medical students don’t really get to know what APRNS are or be forced to include them in language, i.e., ‘when you're seeing your doctor, APRN, or nurse-midwife.’ It felt great to bring representation to my profession and to connect across disciplines. And schools!” (Kwok et al. 2022).

It is a foundational principle of medical humanities that the appreciation of literature and other humanities enrich and expand medical training (Shapiro et al. 2009; Wald et al. 2019). But what are the mechanisms by which this happens? As a faculty member (JH) and a student (TP) who met during the poetry and medicine seminar, this seminar helped us think about this question. Here, we reflect on three ways that we have seen reading poetry applies to medicine: it has the potential to enhance observational skills, model an appreciation of uncertainties, and generate joy.

## Observational skills

Poetry can enrich the care we give by enhancing our observational skills. The role of visual arts in enhancing observational skills and habits has been described in multiple settings (Jasani and Saks 2013). In an 1884 article entitled, “The Art of Fiction,” Henry James encouraged his reader to “try to be one of the people on whom nothing is lost” (James 1884). In medical training, there is an expected focus on the vast content of numerous exams. However, this is distinct from the attentiveness needed to care for patients, which requires a presence to stories and words. Close reading in poetry offers practice in being attentive that can translate into closely listening to patients. Poetry compels one to use a metaphorical magnifying glass, which we can then use to direct our attention to patients. Did we notice their body language? Their subtle tremor? Did we notice a child’s injuries behind the ear and suspect potential child abuse? Did we recognize the splinter hemorrhages on their nails or the Kayser-Fleischer rings in their eyes or the Hawaiian tribal markings on their arms? Did we listen to a patient’s questions, did we hear the fear in their voice or detect an underlying theme of their story?

Close observations and close listening facilitate understanding and connection, improving patients’ trust and satisfaction with care (Berman and Chutka 2016). Whether deeply analyzing poems or being deeply aware with patients, maintaining attention and presence is a skill. Like everything else in medicine, it requires discipline, deliberation, and repetition, with poetry readings offering one context for practice to become someone “on whom nothing is lost.”

## Uncertainty

Clinical medicine is steeped in uncertainty (Hatch 2017), and poetry has parallels. The analysis of poetry often proceeds at a word-by-word pace, and by design, each word, each metaphor, and each phrase will resonate in different ways for different readers. Why this word choice? Why split the line or the stanza here? Why this visual form on the page? How does this part link to the whole? The experience of reading and reflecting on a poem, especially when done in a group, is one of sitting amid these possibilities. There is a strong desire to discover and interpret, which is paired with how large the unknowing is. In medicine, too, the search for “answers” is limited by techniques, sensitivities, and specificities of labs, imaging, tests, and other diagnostic procedures (Dahm and Crock 2022). Even in the face of what is traditionally a position of relative certainty: for example, a tissue diagnosis of a cancer, the implications of that diagnosis for a specific patient and the behavior of that cancer in the future are not entirely predictable. To share a cancer prognosis accurately, gracefully, and tactfully with a patient, we first must be comfortable with incompleteness of our predictive models, the complexity of the literature, and the fact that we can never know conclusively how this one patient in front of us will respond to a specific treatment.

Much of effective communication requires explaining the information we have while acknowledging the inherent uncertainties in our understanding. Do we know how to be suspended in a precarious state? Or, more importantly, how to help accompany a patient in that state? Do we have the humility to become comfortable in these uncertain spaces? As a form where ambiguity and alternative interpretation are fundamental, poetry can help us appreciate the inevitable uncertainties in medicine. The frustration we might feel as students and physicians in the uncertainty of a patient’s diagnosis and prognosis often reflects the anger, sadness, and unsettledness that the patient themself feels. A poem full of ambiguity is a vehicle for us to come closer to that patient’s feeling.

## Joy

I (TP) remember leaving a clerkship one evening and my resident sent me off with the words that if one has leisurely time in medical school, one is doing it wrong, in a tone that was only half joking. He added the instructions that all my time should be spent reading. I silently wondered, reading what? Textbooks, journal articles, my patients’ charts? When I went home, I indeed read, though somewhat smugly: It felt like an invisible, silent act of defiance when after reading more traditional medical material, I opened a well-loved copy of Louise Gluck’s works, which was twice the height from the original purchase from my excessive dog-earing of pages. I indulged in my little “rebellious” act to pause and savor each word, which was something I realized that I didn’t do with medical readings. I didn’t skim like it was a clinical note to *extract*, to *mine* the data, interpretations, and plans. These are important acts in medicine, and they are crucial to the care of patients we see. But at that moment of reading, rather than pulling information out, I allowed the poem to pull me in.

Poems house joy, sadness, anger, grief, loss. But as a student and faculty member, we have experienced that it is the joy that is most prominent, and one intention of reading is simply to have a moment of that childlike delight. The goal of this kind of reading, and indeed of our seminar, is not a practical one and there’s no expectation to *take* anything concrete away from it. And yet an activity that has no apparent concrete utility can possess immense intrinsic value. Paradoxically, that may have implications for our own thriving as clinicians: this joy of reading a poem might help refresh ourselves for the other crucial tasks of medical care.

Part of the joy is the intention to just be in the moment: To sit and be with the words, much as we sit with patients. Incorporating poetry into medical curricula brings an opportunity for medical students to engage in an activity that can model a search for stillness, in the hopes that we’ll recreate that stillness and joy through moments with patients.

## Conclusion

Regardless of *how* poetry is incorporated into medical curricula (Shapiro and Rucker 2003; Wolters and Wijnen-Meijer 2012), our experience teaching and taking a brief Poetry and Medicine seminar has shown us how poetry models complementary approaches to some of the more common modes of thinking in medicine. The development of clinical reasoning, which relies on traditional forms of scientific evidence and can help us develop effective pattern recognition and algorithmic pathways, is one of the central tasks of medical education at every level. Algorithms, for example, serve a necessary purpose by fostering immediate, almost reflexive, associations between individual characteristics, risk factors, and presenting signs with disease processes. We are not arguing to replace these, but wonder about how to augment them. Although linear assessments are integral to medical care, what do we miss when we don’t pause between information input and output?

Poetry allows space to transcend instantaneous thoughts and algorithms’ binaries. The non-reductive approach to reading and reflection lends the opportunity to examine our own subjectivities while also more clearly seeing patients for who they are, where they are, and the uncertainties they may be facing. The most foundational parallel is that we hope to arrive at poetry the way we arrive at patients—ready to closely and carefully listen, observe, lend presence, and engage in the un-layering to find and create endurance, sustenance, solace, and healing.
